# Fisetin Prevents HT22 Cells From High Glucose-Induced Neurotoxicity *via* PI3K/Akt/CREB Signaling Pathway

**DOI:** 10.3389/fnins.2020.00241

**Published:** 2020-03-19

**Authors:** Shenshen Zhang, Ran Xue, Yaping Geng, Hao Wang, Wenjie Li

**Affiliations:** ^1^Precision Nutrition Innovation Center, College of Public Health, Zhengzhou University, Zhengzhou, China; ^2^Department of Nutrition and Food Hygiene, College of Public Health, Zhengzhou University, Zhengzhou, China

**Keywords:** fisetin, HT22 cell, high glucose, neurotoxicity, neuroprotection

## Abstract

Hyperglycemia has been widely considered as a key risk factor for diabetic encephalopathy which can cause neuronal apoptosis and cognitive deficits. The flavonoid compound, fisetin, possesses potential neuroprotective effects and also enhances learning and memory. However, the role of fisetin in hyperglycemia-induced neuronal cytotoxicity has not been fully elucidated. In the present study, HT22 murine hippocampal neuronal cell line was used to establish the injured cell model. Cell proliferation and cytotoxicity assay, Hoechst 33258 staining, qRT-PCR, western blot analysis, and specific inhibitor were used to investigate the effect and molecular mechanisms of fisetin on high glucose (HG)-induced neurotoxicity in HT22 cells. Our results showed that 125 μM and 48 h of treatment was identified as optimal damage parameter of HG. Fisetin significantly improved HG-inhibited cell viability. The levels of LDH, malondialdehyde (MDA), and superoxide dismutase (SOD) were noticeably modulated by fisetin, which alleviated HG-induced HT22 cell oxidative damage. Besides, the apoptosis of HT22 cells was rescued by fisetin pretreatment. In addition, fisetin also prevented HG-induced downregulation of the mRNA expression of *Bdnf*, *Gdnf*, synaptophysin (*Syp*), and glutamate ionotropic receptor AMPA type subunit 1 (*Gria1*) in cells. More importantly, the decreased phosphorylation of phosphoinositide 3 kinase (PI3K), Akt, and cAMP-response element binding protein (CREB) was rescued by fisetin treatment and that neuroprotective effect of fisetin was partially blocked by PI3K inhibitor, LY294002. These findings indicate that fisetin has potent neuroprotective effect and prevents HG-induced neurotoxicity by activation of PI3K/Akt/CREB pathway.

## Introduction

Diabetic encephalopathy is one of the most common crippling complications resulting from diabetes mellitus (DM) affecting central nervous system (Zenker et al., 2013; Simo et al., 2017). Accumulated evidences have demonstrated that DM patients have a significantly higher risk of suffering from cognitive dysfunction (Biessels et al., 2006; Biessels and Despa, 2018). Thus, the importance of diabetic encephalopathy is increasingly being recognized. Currently, loads of researches focus on searching for safe and effective agents to prevent the loss of cognitive function for DM patients.

Several prospective studies have shown that high blood glucose level is a key risk factor in DM-induced cognitive dysfunction and dementia (Kaeidi et al., 2019; Zhang et al., 2019; Sharma et al., 2020). The neuronal glucose level in the central nervous system can be induced up to four-fold increase by hyperglycemia in diabetes (Zhu et al., 2018). The abnormal intracellular glucose metabolism possesses multiple toxic effects on brain, such as formation of advanced glycated end products (AGEs), generation of ROS, and activation of polyol, diacylglycerol, and hexosamine pathways, leading to cognitive dysfunction (Seto et al., 2015). Nonetheless, no specific means is able to protect against the neurotoxicity of hyperglycemia. It is noteworthy that the cellular senescence and apoptosis is a crucial neurotoxicity induced by hyperglycemia (Arunachalam et al., 2014). Thus, therapeutically targeting hyperglycemia-induced damage of nerve cells and explicit the molecular mechanism would be a novel strategy for treating diabetic neuropathy.

The CREB is a transcription regulatory and abundant in brain, particular in neurons. The activation of CREB pathway is closely correlated with the number of surviving neurons, which play important roles in learning and memory in brain (Caracciolo et al., 2018). CREB can also regulate various targeting molecular, including the BDNF, which is essential for neuronal development and survival, synaptic plasticity, and cognitive function (Feng et al., 2019). In neuronal cells, the activation of CREB can be stimulated by the phosphorylation of the upstream PI3K and its effector Akt. Zhang W. et al. (2018) addressed that administration of diterpene ginkgolides could protect against cerebral ischemia/reperfusion damage in rats by upregulating the activation of CREB and Nrf2 through PI3K/Akt signaling pathway. Pretreatment of SH-SY5Y cells with rifampicin remarkably enhanced the phosphorylation of PI3K, Akt, and CREB, exhibiting neuroprotective effects against rotenone-induced apoptosis (Wu et al., 2018). Insulin resistance-induced hyperglycemia downregulated the Akt/CREB signaling pathway caused the obstacle of neuronal pathology in hippocampus neurons and cognitive deficits (Xiang et al., 2015). It should be noted that PI3K/Akt/CREB signaling would be a promising target pathway for treatment of diabetic neuropathies in brain.

**FIGURE 1:**
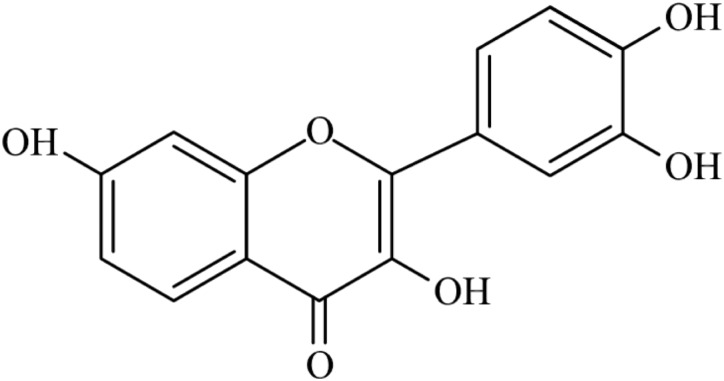
The chemical structural formula of fisetin.

Diabetes mellitus-induced cognitive dysfunction is a great problem for public health. Despite the therapeutic benefits of antidiabetic agents for the treatment of DM-induced cognitive dysfunction, most of these pharmaceutical agents are associated with various undesirable side-effects (Meneses et al., 2015). Recently, medicinal natural extracts appear to offer effective effects in improving DM-related complications with minimal toxicity and side-effects. Fisetin (3,3′,4′,7-tetrahydroxy flavone), a ubiquitous flavonoid, widely exists in strawberries, grape seed, apple, onion, and persimmon (Ma et al., 2018). The chemical structural formula is shown in Figure 1. It has been reported that fisetin possesses neuroprotection potentials, antioxidant, antitumor, anti-inflammation by affecting multiple molecular and signaling pathways (Nabavi et al., 2016; Zhang et al., 2016). Furthermore, fisetin exhibits high brain uptake potential. Krasieva et al. (2015) observed that fisetin can be rapidly detected in the nucleoli of HT22 cells during incubation and in the brain of mice after oral and ip administration. Maher (2009) also found that fisetin exhibits high brain uptake potential. Fisetin can reduce cognitive deficits in old SAMP8 mice while improving impaired synaptic function, age-associated stress, and inflammation (Currais et al., 2018). However, the effect of fisetin on hyperglycemia-induced neurotoxicity has not been fully addressed. In the present study, we investigated the neuroprotective effect of fisetin on HG-induced cell apoptosis in HT22 cells, and whether fisetin protected HT22 cells against HG-induced neurotoxicity *via* PI3K/Akt/CREB signaling pathway would be studied.

## Materials and Methods

### Reagents

Fisetin, glucose and dimethyl sulfoxide (DMSO) were obtained from Sigma–Aldrich (St. Louis, MO, United States). Fisetin (the purity is > 98%) was dissolved in < 0.1% of DMSO solution. LY294002 (the specific inhibitor of PI3K) was bought from MedChemExpress (Shanghai, China). Cell counting kit-8 (CCK-8) was purchased from Dojindo China Co., Ltd. (Shanghai, China). LDH cytotoxicity assay kit, lipid peroxidation [malondialdehyde (MDA)] assay kit, superoxide dismutase (SOD) assay kit, and Hoechst 33258 were obtained from Beyotime (Shanghai, China). Dulbecco’s modified eagle’s medium (DMEM) and phosphate-buffered saline (PBS) were purchased from Biological Industries (Shanghai, China). Fetal bovine serum (FBS) was purchased from Gibco (Grand Island, NY, United States). Antibody against PI3K (#4257), Phospho-PI3Kp85/p55 (#4228), Akt (#4691), Phospho-Akt (#4060), CREB (#4820), and p-CREB (#9198) were obtained from Cell Signaling Technology (Beverly, MA, United States). Antibody against β-actin was purchase from Absin (Shanghai, China).

### Cell Culture

HT22 cells, a mouse hippocampal neuronal cell line, were a gift offered by prof. Deng (Medical School, Hunan University of Chinese Medicine). Optimal growth and survival rate of HT22 cells require 25 mM basal glucose (Fan et al., 2016). Hence, cells were cultivated in HG DMEM medium supplemented with 10% FBS, 100 U/mL penicillin, and 100 mg/mL streptomycin (Solarbio, Beijing, China) in a humidified incubator (5% CO_2_, 37°C).

### Cell Viability Assay

The cell viability of HT22 cells was determined using the CCK-8 assay. Briefly, cells were seeded into 96-well plates at a density of 1 × 10^4^/well for 12 h. After treatment with HG (25–175 mM) or fisetin (0–20 μM) for indicated time, CCK-8 solution was added to each well and incubated for another 2 h in the incubator. Mannitol was osmotic pressure control group. Then, absorbance was measured using a microplate reader (Molecular Devices, San Jose, CA, United States). Cell viability was expressed as the percentage of Abs 570 nm of vehicle control.

### Detection of Superoxide Dismutase (SOD) Activity, Malondialdehyde (MDA) Content, and LDH Leakage Rate

After treatment, HT22 cells were lyzed with lysis buffer (Beyotime Biotechnology, Jiangsu, China) after which SOD activity, MDA content, and LDH leakage rate were detected according to the manufacturer’s instruction and normalized to the total protein content, respectively.

### Hoechst 33258 Staining Assay

To observe the morphological changes in the nuclear chromatin of HT22 cells, the chromatin-specific dye Hoechst 33258 staining was used to stain the nuclei. Cells were seeded in six-well plates at a density of 1 × 10^5^ cells/well and then treated with HG in the presence or absence of fisetin for 48 h. Subsequently, the culture medium was removed, washed thrice with PBS, and then fixed with 4% paraformaldehyde for 20 min. After washed three times with PBS, cells were stained with 50 μg/mL Hoechst 33258 solution for 15 min in the dark. Finally, HT22 cells were observed and photographed using the fluorescence microscope (Nikon, Tokyo, Japan). The apoptosis rate of HT22 cells was quantified by the ratio of cells with fragmented and densely stained nuclei to all cells.

### Quantitative Real-Time PCR (qPCR)

HT22 cells treated with HG in the presence or absence of fisetin were harvested for qPCR. Total RNA was purified from HT22 cells using Trizol reagent (Pufei Biotechnology, Shanghai, China). Total cDNA synthesis was performed using PrimeScript^TM^ RT Master Mix (Takara, Dalian, China) according to the manufacturer’s instructions. QPCR was performed to detect transcript levels of *Bdnf*, *Gdnf*, *Syp*, and *Gria1* using the One Step TB Green^TM^ PrimeScript^TM^ RT-PCR Kit (Takara, Dalian, China). Primer sequences are as follows (Table 1).

**TABLE 1:** Primers sequences used for quantitative PCR.

Gene	Primers
Bdnf	Forward-5′ CGGCCCAACGAAGAAAACCATAA3′, Reverse-5′ GGCGCCGAACCCTCATAGACAT3′
Gdnf	Forward-5′ GATATTGCAGCGGTTCCTG3′, Reverse-5′ CCTGGCCTACTTTGTCACTTG3′
Gria1	Forward-5′ATGTGGAAGCAAGGACTCCG3′, Reverse-5′ CCACATTGCTCAGGCTCAGA3′
Syp	Forward-5′TGTGCCAACAAGACGGAGAG3′, Reverse-5′TTTAACGCAGGAGGGTGCAT3′

### Western Blotting

HT22 cells were collected and total cell lysates were prepared using Phosphorylated Protein Extraction Kit (KeyGen Biotech, Nanjing, China). Equal amounts of protein extracts were mixed (5:1) with loading buffer for electrophoresis in acylamide SDS gels. After electrophoresis, samples were transferred onto polyvinylidene fluoride membrane (Millipore, MA, United States). The proteins were exposed to the specific primary antibodies against the target protein. Cross-reactivity was observed using species-specific secondary antibodies labeled with horseradish peroxidase (HRP) and ECL Plus kit in gel imaging analysis system (Biorad, CA, United States). The quantitative analysis of each bolt was carried out by Quantity One 1-D analysis software (Biorad, CA, United States).

### Statistical Analysis

Statistical analyses were carried out with GraphPad Prism software, version 6.0. All data were presented as the means ± SEM. Differences between the groups were tested by one-way analysis of variance (ANOVA) followed by Student’s test. *P* < 0.05 was considered statistically significant.

## Results

### Effect of Fisetin or HG on HT22 Cells Viability

**FIGURE 2:**
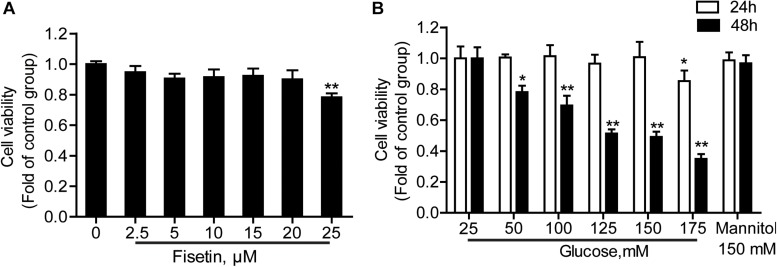
Effect of different concentrations of fisetin or glucoseon HT22 cell viability. **(A)** HT22 cells were incubated with fisetin at different concentrations (2.5, 5, 10,15, 20, 25 μM) for 48 h. **(B)** CCK-8 assay for cell viability in HT22 cells treated with different concentrations of glucose (25, 50, 100, 125, 150, 175 mM). The data are presented as the mean ± SEM, *n* = 4; *P < 0.05, **P < 0.01 *vs.* the 25 mM glucose (control) group.

HT22 cells were treated with a serial concentrations of fisetin (0–25 μM) for 48 h; 25 μM of fisetin reduced cell viability to 78.64 ± 0.48% of that in the control group. At the concentration of below 20 μM (cell viability ≥ 90.05 ± 5.89%), there was no significant difference between fisetin treatment group and control (*P* > 0.05) (Figure 2A). Therefore, lower concentration (0–20 μM) of fisetin was used which was safe and had no influence on the survival of HT22 cells; 25–175 mM of HG (DMEM with HG containing 25 mM glucose) treated HT22 cell for 24 or 48 h, respectively. Mannitol (150 mM) was osmotic pressure control group. The results showed that cell viability was remarkably decreased with the increasing concentration of fisetin at 48 h. Glucose at 125 and 150 mM decreased cell viability to 51.33 ± 2.73 and 49.07 ± 3.41% (*P* < 0.001), respectively (Figure 2B). Thus, 125 mM of HG was a sub-toxic concentration to construct HT22 cell damage model in the subsequent experiments.

### Effects of Fisetin on HG-Induced Cytotoxicity in HT22 Cells

**FIGURE 3:**
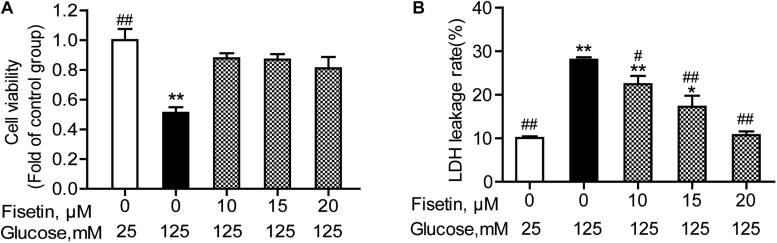
Effect of fisetin on high glucose (HG)-induced neurotoxicity in HT22 cells. HT22 cells were pretreated with fisetin (10, 15, 20 μM) or vehicle control for 1 h followed by high glucose (125 mM) for 48 h **(A)** cell viability is shown in OD value. **(B)** The LDH leakage rate of HT22 cells after treatment with indicated concentration of fisetin and HG. Data were expressed as mean ± SEM of four independent experiments. *P < 0.05, **P < 0.01 *vs.* the 25 mM glucose (control) group; #*P* < 0.05, ##*P* < 0.01 *vs.* 125 mM glucose (HG) group.

To determine whether fisetin could protect neurons from HG injury, HT22 cells were pretreated with fisetin (0–25 μM) for 1 h followed by indicated concentration of HG (125 mM) for 48 h. As shown in Figure 3A, treatment with HG significant inhibited HT22 cells proliferation (51.08 ± 3.74%), while treatment with fisetin strikingly increased cell viability and almost make it recover to the normal level.

The LDH leakage rate was elevated after HG stimulation, and treatment with 125 mM of HG alone increased the LDH leakage rate to 28.04 ± 1.03% (*P* < 0.001), compared to control group. Pretreatment with fisetin, the LDH leakage rate was rescued to base line at 20 μM fisetin (10.7 ± 1.02%, *P* < 0.001) compared to the HG-treated group (Figure 3B). All these results strongly suggested that fisetin could protect HT22 cells from HG-induced cytotoxicity.

### Effect of Fisetin on HG-Induced Oxidative State in HT22 Cells

**FIGURE 4:**
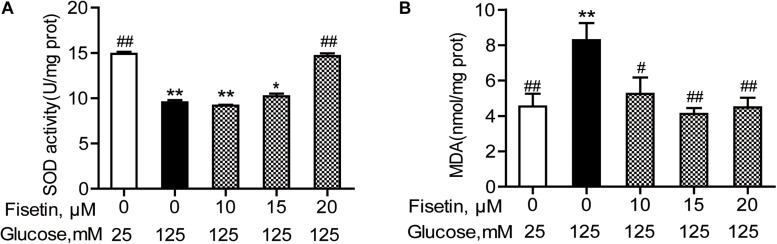
Fisetin alleviates high glucose (HG)-induced oxidative damage in HT22 cells. Cells were pretreated with 10–20 μM of fisetin or vehicle control for 1 h and then exposed to 125 mM of high glucose for 48 h. **(A)** SOD activity and **(B)** MDA content were determined using the test kits. Data were expressed as mean ± SEM, *n* = 4. *P < 0.05, **P < 0.01 *vs.* the 25 mM glucose (control) group; #*P* < 0.05, ##*P* < 0.01 *vs.* 125 mM glucose (HG) group.

The antioxidant activity of fisetin was evaluated, including SOD activity and MDA content. As expected, the results showed that compared with the vehicle control, HG treatment alone significantly inhibited SOD activity to 9.43 ± 0.25 U/mg prot (*P* < 0.001), but increased MDA content to 8.28 ± 0.96 mmol/mg prot (*P* = 0.005) (Figure 4). Whereas fisetin pretreatment dramatically reversed this trend, the SOD activity of 20 μM fisetin was 14.65 ± 0.30 U/mg prot, obviously higher than HG-alone treated group (*P* < 0.001). The MDA contents of fisetin (10, 15, 20 μM) pretreated groups were dramatically suppressed compared with HG group (*P* = 0.016, 0.003, 0.005, respectively). These results showed that pretreatment with fisetin protected HT22 cells against HG-induced cell oxidative damage.

### Fisetin Protects Against HG-Induced HT22 Cells Apoptosis

**FIGURE 5:**
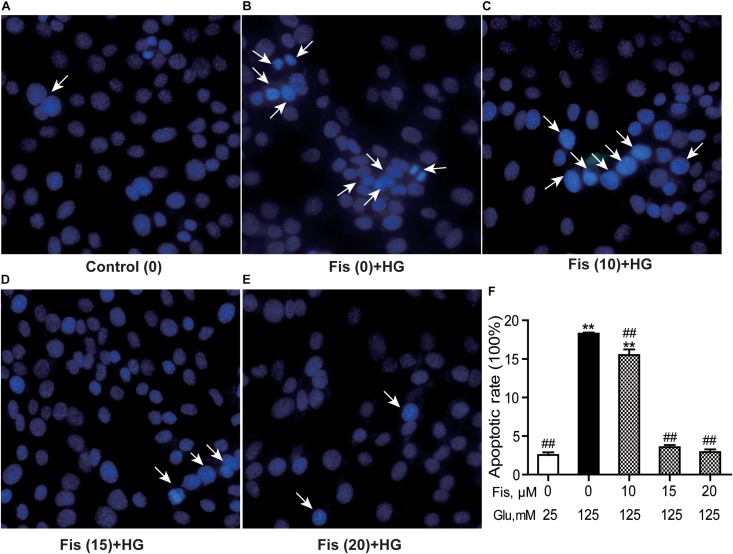
Protective effects of fisetin against high glucose (HG)-induced apoptosis in HT22 cells. HT22 cells were stimulated with high glucose (125 mM) for 48 h in the presence or absence of fisetin. The nuclear structure of each group was stained with Hoechst 33258. **(A)** Control, **(B)** HG, **(C)** Fisetin (10 μM) + HG, **(D)** Fisetin (15 μM) + HG, **(E)** Fisetin (20 μM) + HG. **(F)** Quantitative analysis of high glucose induced apoptosis in each group. Fis: fisetin, Glu: glucose. **P < 0.01 *vs.* the 25 mM glucose (control) group; ##*P* < 0.01 *vs.* 125 mM glucose (HG) group.

Hoechst 33258 staining was used to quantify the levels of apoptosis in each group. As shown in Figure 5, the control group cell apoptosis rate is 2.55 ± 0.45%. HG treatment alone significantly promoted HT22 cells apoptosis (18.28 ± 0.34%) compared to the control group (*P* < 0.001). As expected, pretreatment with fisetin effectively attenuated the apoptosis induced by HG in HT22 cells. Compared with HG group, the cell apoptotic rate was significantly decreased to 2.92 ± 0.49% at 20 μM of fisetin (*P* < 0.001).

### Changes in HG-Induced Gene Expression by Fisetin Treatment

**FIGURE 6:**
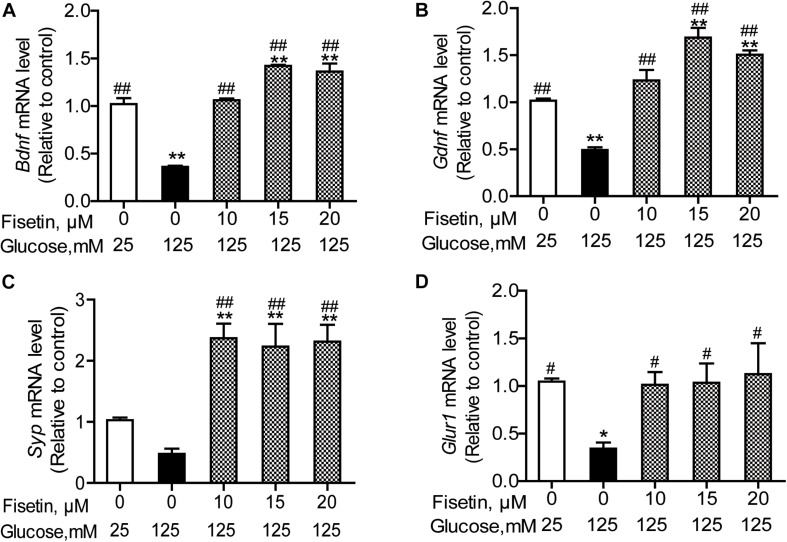
Effect of fisetin on relative mRNA expression levels of Bdnf **(A)**, Gdnf **(B)**, Syp **(C)**, and Gria1 **(D)** in HT22 cells. Cells were preincubated with or without fisetin for 1 h, and then incubated with high glucose (125 mM) for 48 h. *Bdnf*, *Gdnf*, *Syp*, and *Gria1* mRNA levels were examined by quantitative PCR. Data were expressed as mean ± SEM, *n* = 4. *P < 0.05, **P < 0.01 *vs.* the 25 mM glucose (control) group; #*P* < 0.05, ##*P* < 0.01 *vs.* 125 mM glucose (HG) group.

To elucidate the neuroprotective effect of fisetin against HG, we explored the expressions of several neurotrophic factors. As shown in Figure 6, the qRT-PCR results revealed that there was significant decrease in the mRNA expression of *Bdnf*, *Gdnf*, and *Gria1* in HG treated group (0.36 ± 0.01, 0.49 ± 0.028, 0.34 ± 0.06, respectively), compared with control group (*P* < 0.05); *Syp* was also decreased to 0.48 ± 0.08, but not significant (*P* = 0.12). However, when HT22 cells were pretreated with fisetin for 1 h before incubation with HG, the mRNA expression of *Bdnf*, *Gdnf*, *Syp*, and *Gria1* was markedly upregulated in a dose-dependent manner compared with HG group (*P* < 0.05). Fisetin could dramatically neutralize the damage effect of HG on HT22 cells.

### Fisetin Enhances Phosphorylation of CREB, Akt, and PI3K in HG-Induced HT22 Cells

**FIGURE 7:**
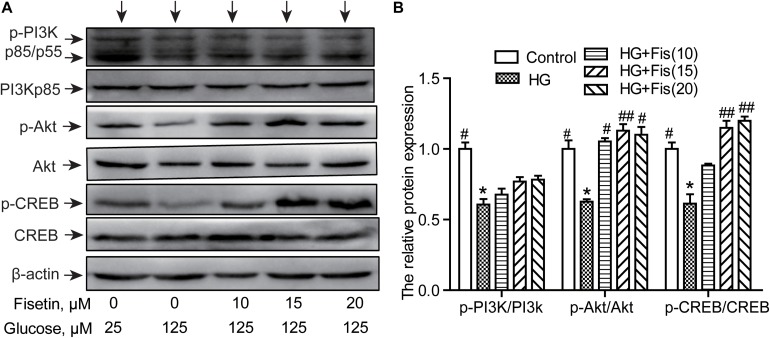
Effect of fisetin on the PI3K/Akt/CREB signaling pathway. **(A)** Western blot analysis of the expression of phosphorylated PI3K, Akt, CREB, and total PI3K, Akt, CREB in HT22 cells. **(B)** The histograms showing their relative expression (phosphorylation was expressed as ratio with respect to their total form). The results were expressed as mean ± SEM (*n* = 3). *P < 0.05, **P < 0.01 *vs.* the 25 mM glucose (control) group; #*P* < 0.05, ##*P* < 0.01 *vs.* 125 mM glucose (HG) group.

To illustrate the molecular mechanisms responsible for the improvement of HG-induced cytotoxicity by fisetin, the activation of PI3K/Akt/CREB pathway was assessed *via* Western blotting assay. As shown in Figure 7, the p-PI3K/PI3K ratio, p-Akt/Akt ratio, and p-CREB/CREB ratio of the control group were set as 1. The relative level of p-PI3K/PI3K, p-Akt/Akt, and p-CREB/CREB inhibited after exposure to HG compared to the control group (*P* < 0.05). However, pretreatment with fisetin obviously rescued the HG-inhibited expressions of p-CREB and p-Akt (*P* < 0.05 and *P* < 0.01). In addition, the expression of p-PI3K exhibited an upward tendency compared with the HG group, but not significant (Figure 7).

### Suppression of the PI3K/Akt/CREB Signaling Pathway Reverses Fisetin’s Neuroprotection in HG-Induced HT22 Cells

**FIGURE 8:**
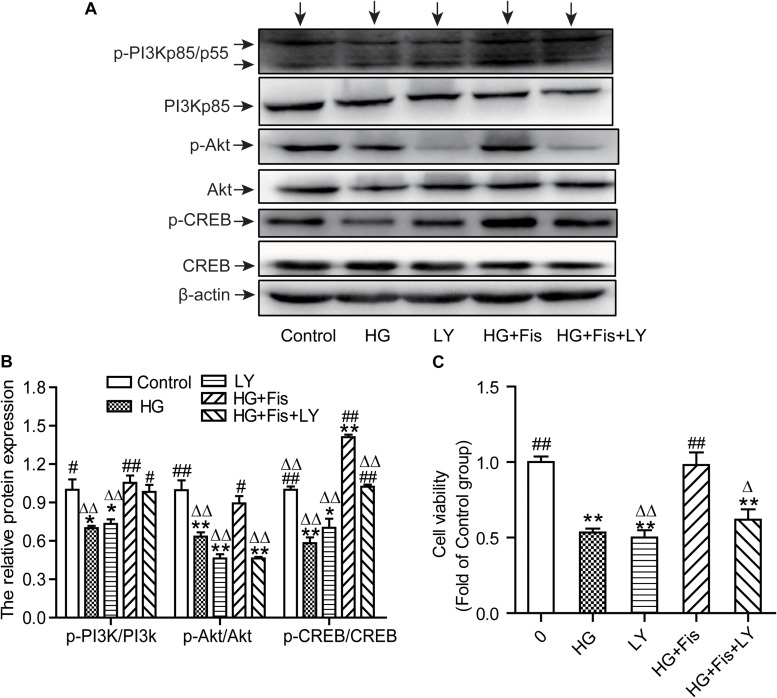
LY294002 blocked the protection of fisetin against high glucose (HG)-induced cytotoxicity. **(A)** Protein expressions were tested. **(B)** Quantitative analysis of the blots was shown in panel after normalized by Quantity One Software. **(C)** Cell viability was determined by CCK-8 assay. Data were expressed as mean ± SEM (*n* = 3). Ctr: normal HT22 cells, HG: HT22 cells exposed to high glucose (125 mM) for 48 h, LY: HT22 cells incubated with LY294002 at 10 μM, HG + Fis: high glucose (125 mM) incubated cells pre-incubated with fisetin (20 μM) for 1 h. HG + Fis + LY: HT22 cells preincubated with LY294002 (10 μM) for 30 min followed by fisetin (20 μM) and high glucose (125 mM). *P < 0.05, **P < 0.01 *vs.* the 25 mM glucose (control) group; #*P* < 0.05, ##*P* < 0.01 *vs.* 125 mM glucose (HG) group; Δ*P* < 0.05, ΔΔ*P* < 0.01 *vs.* HG + Fis group.

It is well known that CREB can regulate memory, learning, and synaptic transmission as well as neuron survival, differentiation, and axon growth in brain (Sun et al., 2017). To further determine whether the protection of fisetin against HG-induced neurotoxicity was through the activation of PI3K/Akt/CREB signaling pathway, we pretreated HT22 cells with LY294002 (10 μM), a specific PI3K/Akt pathway inhibitor, for 30 min before addition of fisetin. As shown in Figures 8A,B, the control group was set as 1, the ratio of p-PI3K/PI3K, p-Akt/Akt, and p-CREB/CREB of HG group was 0.70 ± 0.01, 0.63 ± 0.03, 0.58 ± 0.04, respectively. Pretreatment with fisetin for 1 h followed by HG could markedly improve protein levels of phosphorylated PI3K, Akt, CREB, and cell viability compared to HG-treated HT22 cells (*P* < 0.05). Unfortunately, this improvement effect of fisetin on p-Akt was almost eliminated by the pre-incubated with inhibitor LY294002 (the ratio is 0.46 ± 0.03). Compared to HG + Fis group, the enhancement of p-CREB and cell viability by fisetin was partially suppressed by LY294002 preincubation in HG + Fis + LY (*P* < 0.05). These results indicated that fisetin protects against HG-induced HT22 cell damage partially through PI3K/Akt/CREB signaling pathway.

## Discussion

Diabetes mellitus-associated neuronal dysfunction is one of the most common crippling complications affecting peripheral nerves of diabetic patients. High blood glucose, the characteristics of DM, has been demonstrated to possess negative effects on cognitive function and brain structure. Chronic high blood glucose could alter the substrate transport, lactate homeostasis, and glutamate/glutamine cycling in hippocampus, leading to cognitive deficits (Zhang et al., 2019). Fisetin, a natural flavonoid, is an orally active, novel neuroprotective and cognition-enhancing molecular, possessing high BBB penetration potential. Flavonoid fisetin was screened out from *Rhus verniciflua*, which could protect HT22 cells against glutamate-induced neurotoxicity through attenuating oxidative stress (Cho et al., 2012). Similarly, another study found fisetin prevented fluoride and dexamethasone co-induced oxidative damage in HT-22 cells (Inkielewicz-Stepniak et al., 2012). However, its role in DM-induced cognitive dysfunction has not been explained. The present study was attempted to understand the molecular mechanisms underlying neuroprotective effect of fisetin on HG-treated HT22 cells.

In the present study, we demonstrated that fisetin pretreatment protected against HG-induced neurotoxicity in HT22 cells *via* the activation of PI3K/Akt/CREB signaling pathway. Fisetin dramatically attenuated HG-induced LDH release, MDA overproduction, decreased SOD activity, and cell apoptosis. Inhibition of PI3K/Akt/CREB signaling pathway by PI3K inhibitor LY294002 partially abolished fisetin-provided protection effects on HG-induced cell apoptosis. In general, the present results indicated that fisetin may possess the ability to protect against hyperglycemia-induced neurotoxicity.

In our study, we used HG-exposed HT22 cells as hyperglycemia-induced neurotoxicity model *in vitro*. First, the concentration of HG and fisetin for *in vitro* studies was detected on the basis of cytotoxicity studies. It was found that 125 mM of HG treated HT22 cells for 48 h could decrease cell viability by about 50%. Moreover, the concentration for fisetin was studied on the basis of CCK-8 assay wherein exposure to 20 μM of fisetin for 48 h did not cause any significant change in cell viability of HT22 cells. Therefore, 125 mM of HG was employed as the sub-toxic dose for HT22 cells in the present study. In addition, 20 μM of fisetin would be selected to investigate the neuroprotective effect in the following study. These results confirmed that the HG-induced neurotoxicity model was established successfully.

Tremendous studies have found that fisetin is effective in treatment of diabetic neuropathy, age-related neurodegenerative diseases and brain injury (Sandireddy et al., 2016; Chen et al., 2018; Currais et al., 2018). One important reason may be that fisetin has high brain uptake potential and BBB penetration (Maher, 2009). There is an ongoing debate about whether fisetin can reach the central nervous system that is sufficient to affect brain function. An *in vivo* study using two-photon exited fluorescence imaging indicated that intraperitoneal injection or oral administration of fisetin is rapidly distributed to brain parenchyma (Krasieva et al., 2015). He et al. (2018) demonstrated that oral fisetin exhibits better bioavailability and BBB permeability than structurally related flavonoids, quecetin, luteolin, and myricetin. The unpublished results from Maher’s laboratory indicated that sulfated and/or glucuronidated forms of fisetin reach concentrations of 30 μM in the cerebrospinal fluid with a plasma half life of 8 h in macaques fed a single oral dose of 25 mg/kg⋅bw (Maher, 2017). Hence, fisetin is likely to exhibit excellent BBB penetration and neuroprotection property. We explored the effect of fisetin on neurons in detail. In this study, fisetin played a protective role in HT22 cells against HG-induced neural damages. When HT22 cells were pretreated with fisetin for 1 h, the notable reduced cell viability induced by HG was restored to the basal level and the LDH release was also attenuated. Oxidative stress has been widely considered as a crucial element in the adverse effects of hyperglycemia to various tissues, including neuronal cells. Zhang L. et al. (2018) discovered that fisetin alleviated oxidative stress and BBB distortion after traumatic brain injury through the activation of the Nrf2/ARE pathway. Our study showed that HG increased the oxidative stress through suppressing SOD activity and elevating MDA production. However, fisetin pretreatment dramatically improved SOD activity while alleviating MDA over-production, playing a significant protective role in HT22 cells. This finding indicated that fisetin alleviated oxidant stress in HG-induced cell damage. Additionally, one of the potential mechanisms for hyperglycemia-induced neural cell death is connected with apoptosis that was assessed by many investigations (Namazi Sarvestani et al., 2018; Kaeidi et al., 2019). Taurine reduced HG-induced the number of apoptotic cell *via* Akt/Bad pathway in HT22 cells which further balanced the levels of Bcl-2 and Bax (Wu et al., 2019). Namazi Sarvestani et al. (2018) found that HG induced neurotoxicity and apoptosis in PC12 cells by increasing the protein expression of pro-apoptotic Bax and caspase 3 and decreasing Bcl-2 protein expression. In our experiment, we obtained the consistent results that stimulation of HT22 cells by HG enhanced cell apoptotic rates compared to control group, while fisetin could significantly attenuated HG-induced apoptosis in HT22 cells. It is suggested that anti-apoptosis participates in the protective effects of fisetin against HG-induced neuronal injury.

To confirm the neuroprotective effect of fisetin, the mRNA expression of *Bdnf*, *Gdnf*, *Syp*, and *Gria1* was tested. BDNF is a highly conserved neurotrophin with pivotal role in neuronal survival, neurogenesis, synaptogenesis, and neuroplasticity (Mizui et al., 2016; De Assis et al., 2018). The expression of BDNF gene (*Bdnf*) is closely linked to all aspects of neuronal functioning, including complex processes of cognition (Morgan et al., 2015). The elevation of BDNF is beneficial for improvements in neuroplasticity and lower ratios of cognitive deficits (Phillips et al., 2014). GDNF is associated with the modulation of synaptic plasticity and the formation of neural circuits (Airaksinen and Saarma, 2002). In ischemia/reperfusion rats, Wharton’s jelly-derived mesenchymal stem cells obviously increased gene expression of GDNF and BDNF and improved the functional learning and memory (Abd El Motteleb et al., 2018). In the present study, it was found that *Bdnf* and *Gdnf* gene expression was notably inhibited in HT22 cells cultured with HG, while fisetin pretreatment dramatically upregulated *Bdnf* and *Gdnf* expression, even higher than control group. Besides, it is widely believed that SYP can mediate synaptic structure and play a part in synaptic plasticity through phosphorylation and release of neurotransmitters (Liu et al., 2016). Chronic fluoride exposures reduced SYP expression and induced aberrant changes of GSK-3β/β-catenin signaling, leading to neuronal apoptosis and impaired synaptic plasticity (Jiang et al., 2019). In addition, it is well known that the *Gria1* gene, ionotropic receptor AMPA type subunit 1, is closely associated with depression and status epilepticus. *Gria1* knockout mice exhibit a phenotype relevant for neuropsychiatric disorders, including reduced synaptic plasticity and attentional deficits (Ang et al., 2018). In our research, we found that *Syp* and *Gria1* gene expression was suppressed by HG, but significantly rescued by fisetin pretreatment. It is suggested that the neuroprotective effect of fisetin should be associated with the improvement of neuronal function-related genes expression.

Diabetes mellitus-related cognitive deficits are one of the typical central nervous system complications, while the underlying molecular mechanisms by which hyperglycemia damage cognitive ability are still uncertain. Research has showed that PI3K/Akt signaling pathway acts as a key regulator in neuronal cell death and survival *via* stimulating multiple downstream targets (Zhang W. et al., 2018). 6-Hydroxydopamine (6-OHDA) and rotenone are both commonly used as neurotoxin in the study of PD. Fisetin could alleviate 6-OHDA- or rotenone-induced cytoxicity and oxidative stress in SH-SY5Y cells by activating PI3K/Akt signaling (Watanabe et al., 2018; Rajendran and Ramachandran, 2019). Whether this signaling pathway has any effect in the protection effect of fisetin on hyperglycemia-induced neurotoxicity is not clear. Akt can phosphorylate a number of downstream molecules related to cell survival and proliferation like CREB (Hu et al., 2017). CREB, a transcriptional activator after being phosphorylated, is essential for memory formation, neuronal plasticity, and apoptosis in hippocampal neurons (Kim et al., 2013). Ahmad et al. (2017) discovered that the suppressed p-PI3K, p-Akt, and p-GSK3β expression in Aβ_1__–__42_-treated mice was reversed by fisetin. In the present study, we found the involvement of the PI3K/Akt/CREB signaling in the protection effect of fisetin on HG-induced HT22 cell injury. Exposure to HG alone in HT22 cells was found to significantly decrease the ratio of p-PI3K/PI3K, p-Akt/Akt, and p-CREB/CREB. Fisetin noticeably ameliorated HG-induced deactivation of Akt and CREB, whereas this action was almost blocked by LY294002, the specific inhibitor of PI3K, especially on the activation of Akt. The phosphorylation of CREB and cell viability was partially eliminated. Thus, we speculated that Akt and CREB is the downstream of PI3K and there may be other pathways activating CREB. Taken together, these findings indicated that the neuroprotection of fisetin against HG may partly lie in the reactivation of the PI3K/Akt/CREB signaling.

Fisetin is present in human diet and has benefit to nervous system. In recent years, human clinical trial with fisetin has been performed for brain ischemic stroke with encouraging outcomes (Feng et al., 2019). There is no evidence for either short- or long-term toxicity of fisetin (Currais et al., 2014). These positive outcomes provide foundation for the development of fisetin as new drug and conduct further clinical studies. Although fisetin exhibits rapid absorption and wide distribution into tissues (kidneys, intestines, liver, and brain) and efficiently cross the BBB, its clinical application is mainly limited because of poor water solubility and high lipophilicity (Krasieva et al., 2015; Mehta et al., 2018). Luckily, novel drug delivery systems can improve the activity and overcoming problems associated with fisetin. The fisetin nanoemulsion intraperitoneally injection has a 24-fold increase in fisetin relative bioavailability, without any difference in systemic exposure compared to free fisetin (Ragelle et al., 2012). This technique will definitely help fisetin develop into a new approach for pharmacological treatment for intractable diabetic neuropathy. However, to translate the neuroprotection potential of fisetin to clinical use, well-designed clinical trials along with the reliable analytical markers are required. More detailed and deeper investigation focused toward human trials, optimization of desired physiological responses in targeted patients and molecular targets of fisetin are required. These studies will contribute to the development of fisetin as therapeutics for diabetic encephalopathy in the future.

## Conclusion

In summary, the current study demonstrates that fisetin is able to elevate cell viability, alleviate oxidative damage, inhibit neuron apoptosis, and improve nerve functional parameters in HT22 cells under HG-induced neurotoxicity. The activation of PI3K/Akt/CREB pathway is involved in neuroprotection of fisetin. The present study provides further support that eating more foods rich in fisetin may be beneficial for nervous system. Based on the neuroprotective activity and high BBB permeability, fisetin might be developed into a new approach for pharmacological treatment of intractable diabetic neuropathy. In future research, we plan to develop further *in vivo* studies and clinical trials to reveal the neuroprotection effects and underlying mechanisms of fisetin against HG-induced cognitive dysfunction.

## Data Availability Statement

All datasets generated for this study are included in the article/supplementary material.

## Author Contributions

SZ and RX designed the experiments. HW and WL contributed to the conception of the work, contributed to the figure preparation, and modified the grammar mistakes. SZ, RX, and YG acquired data for cell experiments and analyzed the final results. SZ wrote the first draft of the manuscript. RX and YG wrote sections of the manuscript. All authors contributed to the manuscript revision and approved the submitted version.

## Conflict of Interest

The authors declare that the research was conducted in the absence of any commercial or financial relationships that could be construed as a potential conflict of interest.

## Abbreviations

Akt: protein kinase B
BBB: blood–brain barrier
BDNF: brain derived neurotrophic factor
CREB: cAMP-response element binding protein
GDNF: glial cell line-derived neurotrophic factor
GluR1: glutamate receptor 1
Gria1: glutamate ionotropic receptor AMPA type subunit 1
HG: high glucose
PI3K: phosphoinositide 3 kinase
SYP: synaptophysin.
